# Psychiatric polygenic risk associates with cortical morphology and functional organization in aging

**DOI:** 10.1038/s41398-017-0036-z

**Published:** 2017-12-11

**Authors:** Annie Lee, Mojun Shen, Anqi Qiu

**Affiliations:** 1 Department of Biomedical Engineering, National University of Singapore, Singapore, 117576 Singapore; 2 Singapore Institute for Clinical Sciences, The Agency for Science, Technology and Research, Singapore, 117609 Singapore; 3 Clinical Imaging Research Center, National University of Singapore, Singapore, 117456 Singapore

## Abstract

Common brain abnormalities in cortical morphology and functional organization are observed in psychiatric disorders and aging, reflecting shared genetic influences. This preliminary study aimed to examine the contribution of a polygenetic risk for psychiatric disorders (PRS_cross_) to aging brain and to identify molecular mechanisms through the use of multimodal brain images, genotypes, and transcriptome data. We showed age-related cortical thinning in bilateral inferior frontal cortex (IFC) and superior temporal gyrus and alterations in the functional connectivity between bilateral IFC and between right IFC and right inferior parietal lobe as a function of PRS_cross_. Interestingly, the genes in PRS_cross_, that contributed most to aging neurodegeneration, were expressed in the functioanlly connected cortical regions. Especially, genes identified through the genotype-functional connectivity association analysis were commonly expressed in both cortical regions and formed strong gene networks with biological processes related to neural plasticity and synaptogenesis, regulated by glutamatergic and GABAergic transmission, neurotrophin signaling, and metabolism. This study suggested integrating genotype and transcriptome with neuroimage data sheds new light on the mechanisms of aging brain.

## Introduction

Normal brain aging is a complex process associated with structural and functional alterations. However, such alterations are not exclusive to advancing age but also to psychiatric disorders^1–6^. Covariance of brain alterations in aging and psychiatric disorders may reflect shared genetic influences and underlying common molecular processes. In this study, we aimed to examine the contribution of psychiatric risk genes to aging brain and to identify common biological processes through the use of multimodal brain images, genotypes, and gene transcriptome profiles.

There is substantial evidence on age-related alterations at the levels of individual structures and neural networks. At an anatomical level, prominent cortical thinning in the prefrontal and parietal cortices and relatively sparse cortical thinning in the temporal and occipital cortices are observed in older adults^7–12^. On the other hand, resting-state fMRI (rs-fMRI) is used to examine brain functional organization at a system level based on the synchronization of resting-state blood-oxygen-level-dependent signals among brain regions^13^. Age is associated with decreased functional connectivity among the central hub regions of the brain, specifically the frontotemporal and frontoparietal functional connectivity^14,15^. Inquisitively, remarkably similar trends of age-related cortical thinning and functional connectivity alterations are observed in patients with psychiatric disorders, including schizophrenia, bipolar disorder, major depression disorder etc.^1–6,16–18^. Meta-analysis reveals gray matter loss in the prefrontal cortex converged across multiple psychiatric disorders. In parallel, the common gray matter loss regions form functional networks that are associated with deficits in executive function observed across psychiatric disorders and aging^19,20^. These findings emphasize the importance of morphology of neural substrates and their corresponding functional organization shared across psychopathology and aging.

The common morphological and functional alterations in aging and psychiatric disorders may in part be due to shared genetic influences. Indeed, genome-wide association studies (GWAS) identify common variants associated with brain morphology in older adults^21,22^ and several of these common variants are found to be associated with psychiatric disorders^23,24^. Alternatively, candidate genetic association studies also reveal the moderation role of psychiatric risk genes in the relationship of morphological and functional alterations with age. For instance, genetic variants of COMT val158met and Disrupted in Schizophrenia 1 (DISC1) modulate age-related prefrontal and parietal cortical thinning^25^. Interestingly, the functional connectivity between the prefrontal and parietal regions is also manipulated by genetic variants of COMT and DISC1^26–30^. Nevertheless, candidate gene approaches or GWAS bear weaknesses and gene products operate in networks, such that alterations in neural systems that increase vulnerability for psychopathology derive from genomic variants at multiple sites and may converge to influence common biological systems. Indeed, psychiatric disorders and aging are polygenic traits in nature^31,32^. The genetic susceptibility to psychiatric disorders and aging appears to reflect the cumulative influence of multiple genetic variants^33^. This idea leads to the use of methods of genomic risk profiling to examine the influence of genetic burden as reflected by a set of “risk” alleles for across psychiatric disorders^34^. Existing GWAS for cross psychiatric disorders (Cross-Disorder Group of the Psychiatric Genomics Consortium, 2013) make it feasible to access the risk alleles and effect sizes of SNPs and facilitate the computation of polygenic risk for characterizing accumulative genetic risks for cross psychiatric disorders. Inquisitively, psychiatric-related genes are also over-represented in the gene set related to aging^35^. This concordance therefore emphasizes the significance of studying polygenic architecture across multiple psychiatric disorders to understand its role and biological processes in aging.

In this study, we aimed to compute a polygenic risk score of cross psychiatric disorders (PRS_cross_) for each individual in a Chinese sample comprised of adults aged 21 years and above, as the sum of the count of risk alleles weighted by the effect size in the discovery sample obtained from existing GWAS on cross psychiatric disorders (Cross-Disorder Group of the Psychiatric Genomics Consortium, 2013). We then aimed to examine whether PRS_cross_ moderates age effects on cortical thickness and its parallel functional organization assessed using both structural and rs-fMRI. Based on the aforementioned genetic influences on the brain in psychiatric disorders and aging, we hypothesized that PRS_cross_ would modulate associations of age with cortical thickness, particularly in the prefrontal and parietal cortex, and functional connectivity between these two regions.

Furthermore, we aimed to identify genes involved in the computation of PRS_cross_ that were most contributed to the modulation effects on age-related alterations in cortical thickness and its functional organization and explore their biological processes. Recent studies observed the similarity of the cortical topological organization identified based on rs-fMRI and transcriptome data^36,37^, suggesting the potential use of imaging genetics (combination of genotype and rs-fMRI) for understanding molecular basis supporting cortical functional organization. In this study, we further took advantage of the available brain transcriptome database^38^ and aimed (1) to identify genes with common gene expression profiles in the cortical regions whose thickness or functional connectivity was as a function of age by gene interaction; (2) to investigate biological processes formed by this set of genes using weighted gene co-expression network analysis (WGCNA)^39^ and gene ontology enrichment analysis. The transcriptome analysis evaluated validity of the age by gene interaction effects on the cortical morphology and functional organization. Overall, covariance in biological processes found by genotype–brain associations and the unique transcriptional signature of the cortical morphology and cortico-cortical organization may provide convergent understanding of biological processes in aging.

## Material and methods

### Subjects

Two hundred and fourteen healthy Singaporean Chinese volunteers aged from 21 to 80 years old were recruited (males: 93; females: 121) for this study. Volunteers with the following conditions were excluded: (1) major illnesses/surgery (heart, brain, kidney, lung surgery); (2) neurological or psychiatric disorders; (3) learning disability or attention deficit hyperactive disorder; (4) head injury with loss of consciousness; (5) non-removable metal objects on/in the body such as a cardiac pacemaker; (8) diabetes or obesity; (9) a MMSE score of less than 24^40^. This study was approved by the National University of Singapore Institutional Review Board and all participants provided written informed consent prior to participation.

### MRI data acquisition

MRI scans were acquired using a 3 T Siemens Magnetom Trio Tim scanner with a 32-channel head coil at the Clinical Imaging Research Centre of the National University of Singapore. The image protocols were (i) high-resolution isotropic T_1_-weighted Magnetization Prepared Rapid Gradient Recalled Echo (MPRAGE; 192 slices, 1 mm thickness, sagittal acquisition, field of view 256 × 256 mm, matrix = 256 × 256, repetition time = 2300 ms, echo time = 1.90 ms, inversion time = 900 ms, flip angle = 9°); (ii) isotropic axial resting-state functional MRI imaging protocol (single-shot echo-planar imaging; 48 slices with 3 mm slice thickness, no inter-slice gaps, matrix = 64* × *64, field of view = 192 × 192 mm, repetition time = 2300 ms, echo time = 25 ms, flip angle = 90°, scanning time = 8.01 min). During the rs-fMRI scan, the subjects were asked to close their eyes. The image quality was checked through visual inspection after the acquisition while subjects were still in scanner. If the motion of the image was large, the scan was repeated. However, the image was removed from the study if the motion of the image remains large after 3 repetitions.

### MRI data preprocessing

#### Structural MRI analysis

In the structural *T*_1_-weighted MRI analysis, gray matter, white matter, cerebral spinal fluid (CSF), lateral ventricles, and subcortical structures were automatically segmented from the intensity-inhomogeneity corrected *T*_1_-weighted MR images^41^. For cortical thickness analysis, an inner surface was constructed at the boundary between white and gray matter and then propagated to an outer surface at the boundary between gray matter and CSF. Cortical thickness was measured as the distance between the corresponding points on the inner and outer surfaces^42^. A cortical surface mapping algorithm, large deformation diffeomorphic metric mapping (LDDMM), was then applied to align individual cortical surfaces to an atlas cortical surface for group analysis of cortical thickness^43^.

#### Rs-fMRI network analysis

The rs-fMRI data were first processed with slice timing, motion correction, skull stripping, and grand mean scaling of the data (to whole brain modal value of 100). Within each subject, the rs-fMRI images were aligned to the T_1_-weighted image. six parameters of head motion and CSF and white matter signals were regressed out from the rs-fMRI signals. The functional signals were then band-pass filtered (0.01–0.08 Hz). Finally, the fMRI data were represented on the cortical surface (Qiu et al. 2006) and transformed to the atlas cortical surface based on the LDDMM transformation mentioned above. To quantify the quality of rs-fMRI data in terms of head motion, framewise displacement due to motion averaged over the image volume was calculated for individual subjects. Its mean and standard deviation were respectively 0.057 and 0.033 mm among all the subjects used in this study. The head motion was independent of age (*p* > 0.05).

#### Genotyping and PRS_cross_ computation

Saliva was collected from each participant through the Oragene DNA Self-Collection Kit (DNA Genotek Inc., Kanata, Ontario, Canada). The samples were genotyped on Illumina Omni express arrays, recently shown to perform well and have better coverage than competitors in Asian populations^44^ and on Illumina Exome arrays as well as Taqman array, following the manufacturer’s instructions by Expression Analysis Inc. Data were processed in GenomeStudio Genotyping Module^TM^. Genotyping calls were made by the GenCall software which incorporates a clustering algorithm (GenTrain) and a calling algorithm (Bayesian model). The GenCall score of each SNP probe was generated to rank and filter out failed genotypes^45^. In this study, the genotypes with a GenCall score of less than 0.15 were not assigned genotypes^45^. All SNPs in this study did not deviate from the Hardy-Weinberg equilibrium (HWE, *p* < 0.001)^46^.

The PRS_cross_ was computed based on genotyping data of the subjects in this study and meta-analysis results from the Psychiatric Genomics Consortium (PGC) using plink (Cross-Disorder Group of the PGC, 2013) The PRS_cross_ was a cumulative summary score computed as the sum across the allelic scoring system (0, 1, and 2) of the SNPs of individual weighted by odd ratio (OR) of the risk SNPs. ORs were obtained from the meta-analysis on GWAS on cross-psychiatric disorders (autism spectrum disorder, attention deficit-hyperactivity disorder, bipolar disorder, major depressive disorder, and schizophrenia; http://www.med.unc.edu/pgc). The risk SNPs were selected at several p-value levels (0.01, 0.05, 0.10, 0.15, and 0.20). The resulting numbers of SNPs for PRS_cross_ were 8645, 30518, 54353, 76383 and 97821, respectively. The PRS_cross_ were then standardized to a mean of zero and standard deviation of one.

### Statistical analysis

Age by PRS_cross_ interaction on cortical thickness and rs-fMRI functional connectivity was performed using Surfstat^47^.

#### Cortical thickness

Regression analysis was used to examine age by PRS_cross_ interaction on cortical thickness, where age, PRS_cross_ and the interaction of their mean-centered measures were included as main factors and gender as a covariate. Age was considered as a continuous variable. Results at each surface vertex were thresholded at a level of significance (*p* < 0.005) and were corrected for multiple comparisons at a cluster level of significance (*p* < 0.05). In post hoc analysis, age effects on thickness were examined in low and high genetic risk groups. For this, the sample was divided into two groups, PRS_cross_ < 0 (low) and PRS_cross_ > 0 (high). Thickness was averaged in each cortical cluster with significant age by PRS_cross_ interaction and considered as dependent variable in post hoc regression analysis. Age was used as main factor and gender as covariate. Slope analysis was further employed to compare the slopes of age-related decline in cortical thickness between the two PRS_cross_ groups. The second post hoc analysis examined whether the PRS_cross_ plays the same role in young (20–39 years old), middle (40–54 years old), and old adults (55 and above). Pairwise group comparison analysis was performed on thickness with the PRS_cross_ as a main factor in each age group. All post hoc analyses were run separately for individual brain regions.

#### Resting state fMRI (rs-fMRI)

To examine age by PRS_cross_ interaction on the functional connectivity of the cortical regions identified above, seed-based correlation analysis was used to generate the functional connectivity map of these cortical regions. One-sample Student’s *t-*test was then used to create a group-level functional connectivity map where each surface vertex was thresholded at a level of significance (*p* < 0.005) and then corrected for multiple comparisons at a cluster level of significance (*p* < 0.05).

Subsequently, regression models were used to examine age by PRS_cross_ interaction on the functional connectivity maps obtained from the seed-based correlation analysis. Age, PRS_cross_ and their interaction were included as main factors and gender as a covariate. Age was considered as a continuous variable. Results at each surface vertex were thresholded at the level of significance (*p* < 0.005) and corrected for multiple comparisons at the cluster level of significance (*p* < 0.05). The aforementioned post hoc analyses on thickness were also carried out for the functional connectivity with significant age by PRS_cross_ interaction.

#### Analysis of biological processes for cortical morphology

Our study aimed to identify underlying biological processes for age by PRS_cross_ interaction on cortical thickness. For this, gene transcriptome data of six healthy adult human brains were first extracted from the Allen Brain Atlas database (http://www.brain-map.org) in the brain regions whose cortical thickness was shown with age by gene interaction in this study. Second, SNPs (top 10%) involved in the PRS_cross_ computation that most contributed age by gene interaction on thickness and were then mapped to genes using the batch query function in UCSC Genome Bioinformatics (http://genome.ucsc.edu). Third, this set of genes that express in a particular cortical region was identified and was examined whether they are representative over the set of genes obtained from the age by gene interaction analysis using hypergeometric testing. Fourth, weighted gene co-expression network analysis, a systems biology analysis method, (WGCNA^39^), was used to identify modules of co-expressed genes that correspond to shared functions, such as biological processes. To determine the robustness and reproducibility of each genetic module and whether the module obtained is significantly better than a random sample of genes, module preservation statistical analysis from WGCNA package was carried out^39^. Two types of network-based module preservation statistics were computed, namely (1) module density–based statistic that determines whether genes remain highly connected in the test set and (2) connectivity-based statistic that determines the extent to which connectivity patterns between genes in the test set remain similar when compared with the training set. In each preservation statistic, *Z* statistics that followed standard normal distribution $\left( {{\mathrm{Z}} = \frac{{observed - mean_{permutated}}}{{sd_{permuted}}}} \right)$ was computed from the permutation test. Subsequently, individual *Z* scores were summarized into a composite measure called Preservation *Z*-score. Preservation *Z*-score greater than 10 indicates strong evidence for the module preservation in the test data set while Preservation Z-score less than 2 indicates no evidence of the module preservation. Enrichment analysis on Gene Ontology (GO) terms was further used to explore biological processes of each gene module and Fischer’s exact *p*-value (FDR corrected) was reported.

#### Transcriptome and gene differential expression analysis

In this study, we assumed that genes with common expression profiles between two cortical regions provide molecular basis for the functional organization of these regions. The gene expression data were extracted for the brain regions whose functional connectivity was significantly influenced by age by PRS_cross_ interaction. For a given gene with multiple probes, the expression values were averaged across the probes. A total of 1505 genes of 22 samples in the left IFC and 1505 genes of 6 samples in the right IFC were curated for right IFC-left IFC functional connectivity, and 1150 genes of 6 samples and 1150 genes of 11 samples in the right IPL were curated for right IFC-right IPL functional connectivity. MaxT in bioconductor R package^48^ was used to identify the subset of the above extracted genes with no significant differential expression between two brain regions. To further verify whether the genes selected by maxT have similar gene expression patterns in these two brain regions, Kolmogorov–Smirnov test based on mean (KS test) and variance (RKS test) were employed to test for no presence of differences in shift and no differences in variance or scale of the gene set in two brain regions^49,50^. Moreover, hypergeometric test was used to examine whether this commonly expressed gene set is representative over the gene set that most contributed to age by gene on the functional connectivity of these two brain regions. If this gene subset is over-representative, their biological processes were further explored using WGCNA and GO enrichment analysis as described above.

## Results

### Demographics

Among 214 recruited subjects, 30 subjects with no genetic data and 32 with missing structural MRI or rs-MRI data were excluded, resulting in a final sample size of 174 in this study. Table 1 lists the distribution of age, sex, education level and the Mini-Mental State Examination (MMSE) score in each decade.

**Table 1:** Demographics

*Age Range*	*20s*	*30s*	*40s*	*50s*	*60s*
	*(n=34)*	*(n=20)*	*(n=24)*	*(n=42)*	*(n=51)*
	mean (SD)	mean (SD)	mean (SD)	mean (SD)	mean (SD)
Female, %	47.1	55.0	66.7	57.1	58.8
Age	24.9 (1.78)	34.0 (2.21)	44.2 (2.90)	54.6 (3.12)	67.6 (4.55)
Education Level	4.62 (0.55)	4.65 (0.59)	3.49(1.10)	3.12 (1.06)	2.51 (1.57)
MMSE scores	29.2(0.97)	28.5 (1.65)	28.1(1.28)	28.1 (1.52)	27.6 (1.92)

*Note:* Education Level: 0 = no education, 1 = primary school level, 2 = secondary school level, 3 = Singapore – Cambridge General Certificate of Education Ordinary Level (“O” level) / Singapore- Cambridge General Certificate of Education Normal (Academic) Level (“N” level), 4 = Pre-University/Diploma/ITE/Certificate, 5 = Degree and above

### Age by PRS_cross_ on cortical morphology

**Fig. 1:**
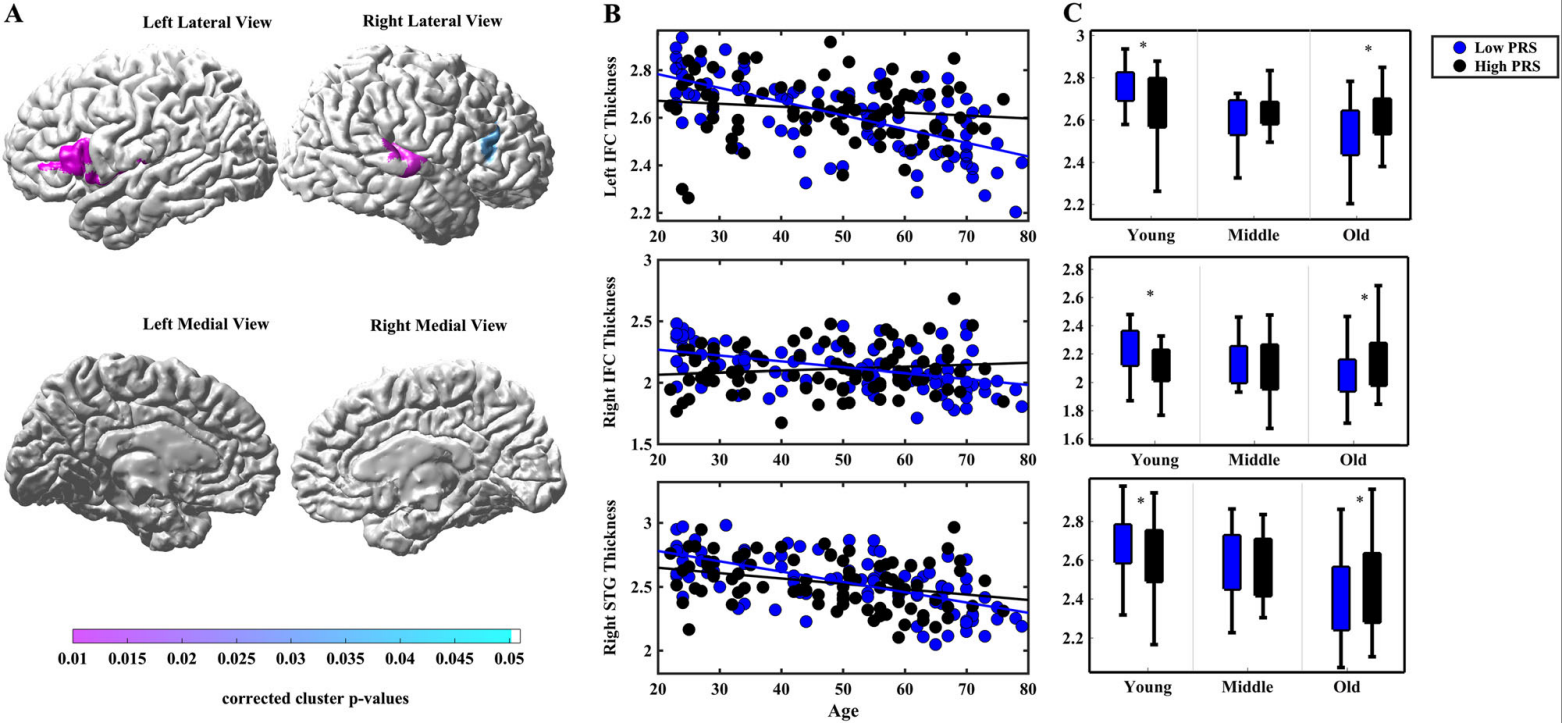
Statistical maps of age by PRS_cross_ interaction on cortical thickness. Panel **a** shows statistical maps of age by PRS_cross_ interaction on cortical thickness. Panel **b** illustrates scatter plots for the relationship of age and cortical thickness in low PRS_cross_ (<0) and high PRS_cross_ (>0) groups. Panel **c** illustrates boxplots of cortical thickness between low and high PRS_cross_ groups in each age group respectively. * *p* < 0.05

Regression analysis was applied to examine age by PRS_cross_ interaction on cortical thickness after adjusting for gender. Age by PRS_cross_ effects on cortical thickness were observed predominantly in the left inferior frontal cortex (IFC) (corrected cluster *p* < 0.001), right IFC (corrected cluster *p* = 0.035) and right posterior region of superior temporal gyrus (STG) (corrected cluster *p* = 0.002) (Fig. 1a) with the largest interaction effect at PRS_cross_ of *p* = 0.01 among the five thresholds (0.01, 0.05, 0.10, 0.15, and 0.2). Post-hoc analysis was carried out to further examine the effect of age on cortical thickness of the aforementioned brain regions in low and high genetic risk groups respectively. Age was found to be negatively associated with cortical thickness in the bilateral IFC and posterior region of STG in the low PRS_cross_ (<0) group. The high PRS_cross_ (>0) group showed that age was negatively associated with cortical thickness in the right posterior segment of STG but not the bilateral IFC (Table 2 **;** Fig. 1b). Slope analysis showed a faster decline in age-related cortical thinning of the bilateral IFC and right posterior segment of STG in the low PRS_cross_ group as compared to the high PRS_cross_ group (column 4 in Table 2 **)**.

**Table 2:** Age by PRS_cross_ interaction on cortical thickness and functional connectivity

	Age by PRS_cross_		Post hoc analysis association of age and brain regions	Slope Analysis	Post hoc analysis in each age group
Brain regions	Cluster size	P-value*	β (P-value)	β (P-value)	P-value
**Cortical Thickness**					
Left IFC	3382	<0.001	Low PRS_cross_ group: −0.614 (<0.001) High PRS_cross_ group: −0.106 (0.345)	−0.918 (0.001)	Young Adults: Low PRS_cross_ > High PRS_cross_; *p* = 0.031 Middle-Aged Adults: Low PRS_cross_ < High PRS_cross_; *p* = 0.212 Older Adults: Low PRS_cross_ < High PRS_cross_; *p* = 0.010
Right IFC	1175	0.035	Low PRS_cross_ group: −0.436 (<0.001) High PRS_cross_ group: 0.079 (0.481)	−0.867 (<0.001)	Young Adults: Low PRS_cross_ > High PRS_cross_; *p* = 0.017 Middle-Aged Adults: Low PRS_cross_ > High PRS_cross_; *p* = 0.301 Old Adults: Low PRS_cross_ < High PRS_cross_; *p* = 0.059
Right posterior segment of STG	2247	0.002	Low PRS_cross_ group: −0.328 (0.003) High PRS_cross_ group: −0.622 (<0.001)	−0.514 (0.032)	Young Adults: Low PRS_cross_ > High PRS_cross_; *p* = 0.051 Middle-Aged Adults Low PRS_cross_ > High PRS_cross_; *p* = 0.060 Old Adults: Low PRS_cross_ < High PRS_cross_; *p* = 0.043
**Functional Connectivity**					
Right IFC-Left-IFC	836	0.024	Low PRS_cross_ group: −0.307 (0.004) High PRS_cross_ group: 0.069 (0.537)	−0.688 (0.010)	Young Adults: Low PRS_cross_ > High PRS_cross_; *p* = 0.047 Middle-Aged Adults: Low PRS_cross_ < High PRS_cross_; *p* = 0.105 Old Adults: Low PRS_cross_ > High PRS_cross_; *p* = 0.652
Right IFC-Right IPL	1284	0.016	Low PRS_cross_ group: −0.401 (<0.001) High PRS_cross_ group: 0.095 (0.397)	−0.937 (<0.001)	Young Adults: Low PRS_cross_ > High PRS_cross_; *p* = 0.012 Middle-Aged Adults: Low PRS_cross_ > High PRS_cross_; *p* = 0.644 Old Adults: Low PRS_cross_ < High PRS_cross_; *p* = 0.425

*IFC* inferior frontal cortex, *STG* superior temporal gyrus, *IPL* inferior parietal lobe

The columns respectively list brain regions with significant age by PRS_cross_ interaction, clusters size and *p*-values, *β* and *p*-values for the associations of age and brain regions among low PRS_cross_ (<0) and high PRS_cross_ (>0) groups, slope analysis for comparing β-values between low and high PRS_cross_ groups, and P-values for post hoc analysis in individual age groups

*Corrected cluster *p*-value

Figure 1c shows boxplots of cortical thickness between the low and high PRS_cross_ groups in young, middle, and old adults. Among the young adults (20–39 years old), the low PRS_cross_ group showed thicker cortex in the bilateral IFC and right posterior segment of STG (marginally significant) as compared to the high PRS_cross_ group. Among the middle-age adults (40–54 years old), there was no significant difference between the low and high PRS_cross_ groups in all the three brain regions (*p* > 0.05). In contrast, among the old adults (55 and above), the low PRS_cross_ group showed thinner cortex in the left IFC, right IFC (marginally significant) and right posterior segment of STG as compared to the high PRS_cross_ group (column 5 in Table 2 **)**.

### Age by PRS_cross_ on functional connectivity

**Fig. 2:**
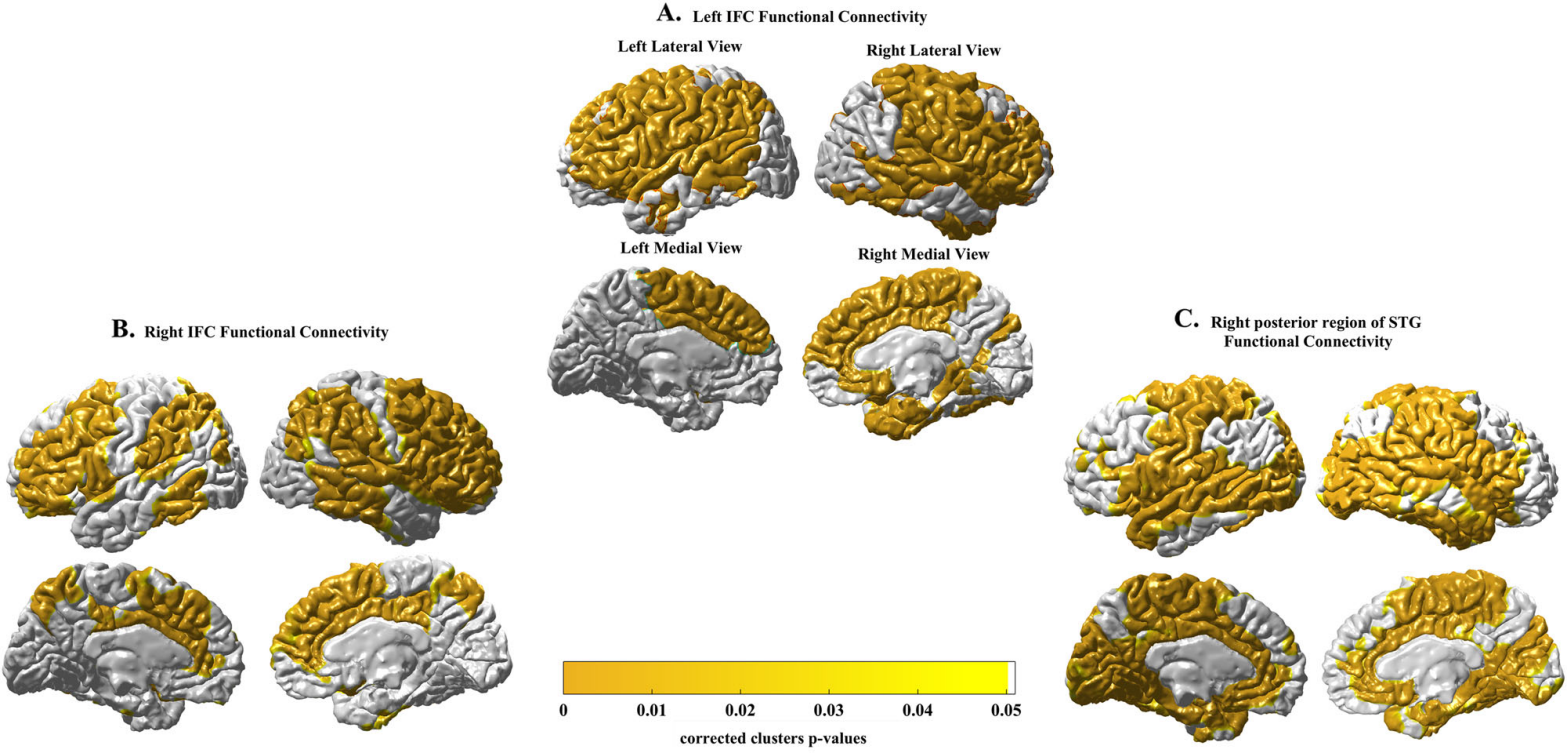
Function connectivity maps of left inferior frontal cortex (IFC), right IFC and right posterior segment of superior temporal gyrus (STG) as shown in Fig. 1a

Recent studies suggest that brain regions with age-related gray matter loss and disruptions in their functional connectivity contribute to age-related deficits in executive function^19,20^. We further examined whether PRS_cross_ moderated age effects on the functional connectivity of the cortical regions identified above. Figure 2 shows the functional connectivity maps of the bilateral IFC and right posterior segment of STG. The left IFC functionally connected with the bilateral superior frontal gyrus (SFG), middle frontal gyrus (MFG), IFC, precentral gyrus, postcentral gyrus, insula, STG, posterior segment of middle temporal gyrus (MTG), anterior cingulate (ACC) as well as posterior cingulate cortex (PCC) and temporal pole of the right hemisphere (Fig. 2a). Likewise, the right IFC showed functional connections with the bilateral STG, MFG, IFC, posterior region of STG and MTG, inferior parietal lobe (IPL), insula, PCC and precuneus (Fig. 2b). The right posterior region of STG had functional connections with the bilateral IFC, bilateral SFG, IFC, precentral gyrus, postcentral gyrus, STG, MTL, ACC, PCC, occipital and inferior temporal lobe of the right hemisphere (Fig. 2c).

**Fig. 3:**
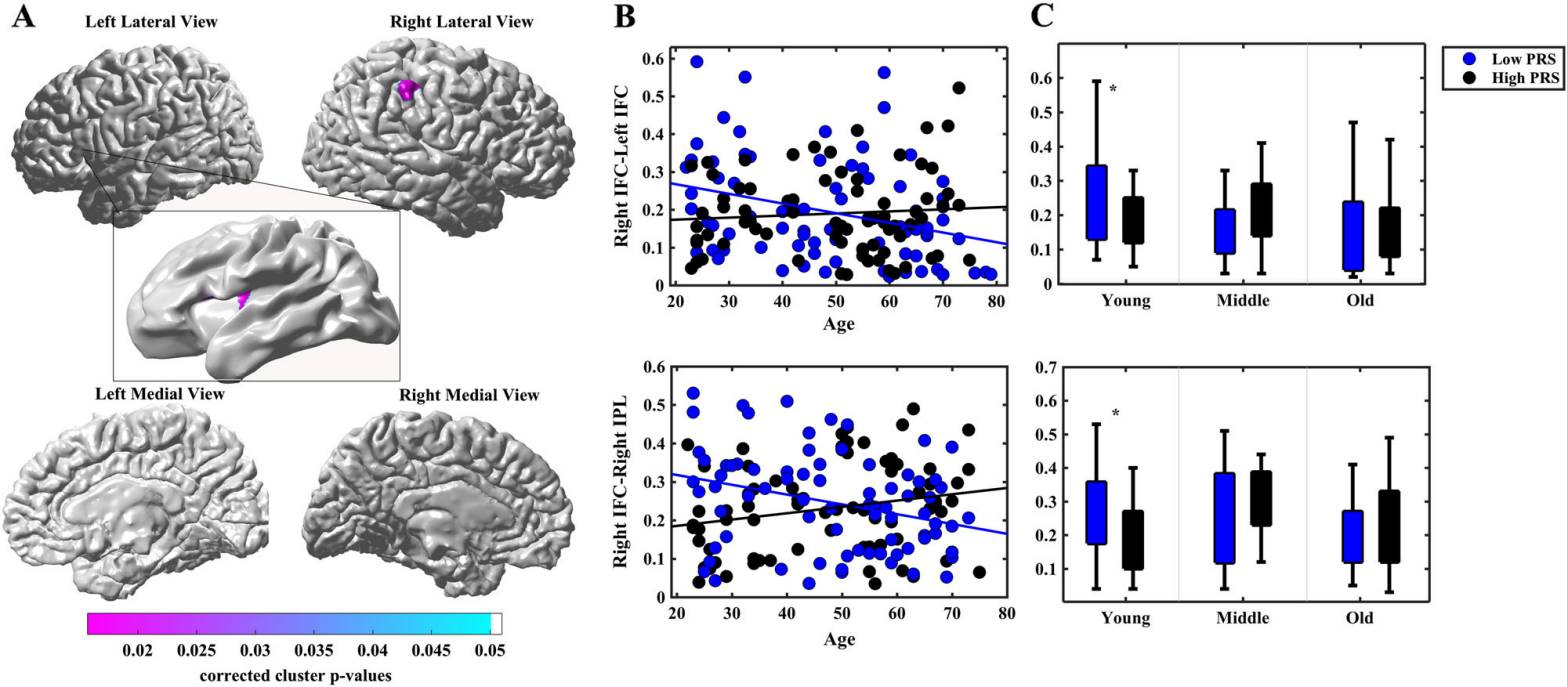
Statistical maps for age by PRS_cross_ interaction on the functional connectivity of right inferior frontal cortex (IFC). Panel **a** shows statistical maps for age by PRS_cross_ interaction on the functional connectivity of right inferior frontal cortex (IFC). Panel **b** shows scatter plots for the relationship of age and functional connectivity of right IFC-left IFC and right IFC-right inferior parietal lobe (IPL) in low PRS_cross_ (<0) and high PRS_cross_ (>0) groups. Panel **c** illustrates boxplots of right IFC-left IFC and right IFC-right IPL functional connectivity in low PRS_cross_ (<0) and high PRS_cross_ (>0) groups of each age group, respectively. *p *< *0.05

Regression analysis was subsequently used to examine whether PRS_cross_ moderates age effects on the above functional connectivity maps. Figure 3a illustrated age by PRS_cross_ interaction on the functional connectivity of right IFC-left IFC (corrected cluster *p* = 0.024) as well as between right IFC-right inferior parietal lobe (IPL) (corrected cluster *p* = 0.016) with the greatest interaction effects at PRS_cross_ of *p* = 0.15 and *p* = 0.10 among the five thresholds (0.01, 0.05, 0.10, 0.15 and 0.2), respectively. Further post-hoc analysis show5ed that in the low PRS_cross_ group, age was negatively associated with the functional connectivity of right IFC with left IFC and right IPL. In contrast, the high PRS_cross_ group did not show any association between age and the functional connectivity of right IFC-left IFC and right IFC-right IPL. Slope analysis showed a faster rate of decline in the functional connectivity of right IFC-left IFC and right IFC-right IPL in the low PRS_cross_ group as compared to the high PRS_cross_ group (column 4 in Table 2 **;** Fig. 3b **)**.

Figure 3c shows boxplots of the functional connectivity of the two PRS_cross_ groups in young, middle, and old adults. Among the young adults, the low PRS_cross_ group showed stronger functional connectivity between right IFC-right IFC and right IFC-right IPL compared to the high PRS_cross_ group. Among the middle and old-age groups, there was no significant difference between the two PRS_cross_ groups in the functional connectivities of right IFC-right IFC and right IFC-right IPL (*p* > 0.05) (Table 2 **column 5)**.

### Biological processes for age by gene interaction on cortical thickness

**Fig. 4:**
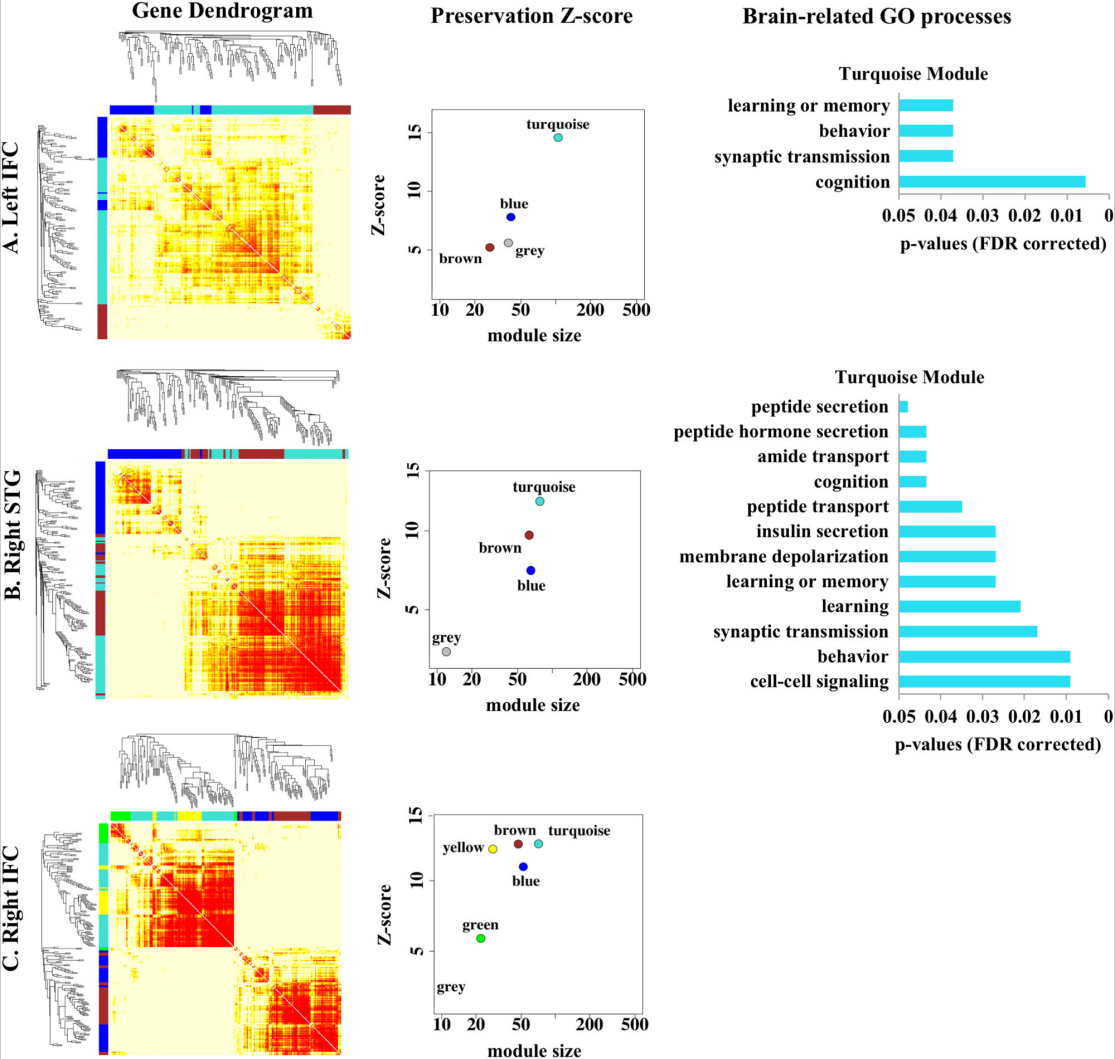
Genetic modules for age by PRS_cross_ interaction on cortical thickness. The first column shows the WGCNA gene dendrogram for left inferior frontal cortex (IFC; top), right superior temporal gyrus (STG; middle), and right IFC. The second column illustrates the robustness of each module as indicated by the Preservation *Z*-score. The third column lists biological processes of the selected module with significant Fischer’s exact p-value (FDR corrected)

Among the SNPs involved in the PRS_cross_ computation, top 10% SNPs (864) that contributed most to age by PRS_cross_ on thickness of left IFC, right IFC and right posterior region of STG were respectively mapped to 259, 265, and 268 genes. For the left IFC, 212 among the 259 genes were expressed in the same cortical region, which were significantly over-representative over the 259 genes (*p* < 0.001). These 212 genes formed four robust modules using WGCNA (Fig. 4a **;** Preservation *Z*-scores ≥ 5.6), namely, turquoise, blue, grey, and brown modules. Among the 4 modules, the turquoise module was the most robust module (Preservation *Z*-score = 15.0). Genetic Ontology (GO) analysis based on Fisher’s exact test (FDR corrected) showed that the turquoise module largely reflected brain related biological processes, such as behavior, cognition, learning or memory and synaptic transmission (Fig. 4a). The major hub genes in this module included GRIN2A, CACNA1A, CTNND2, PLCO, PRKCB, and etc (Table 3). GO biological processes for the other three modules were less related to brain functions and did not survive for the FDR correction of multiple comparisons (Table S1 in the Supplementary Material).

**Table 3:** Genetic modules and their biological processes most relevant to the brain function

Module	Preservation Z-score	Biological Processes	Genes
**Left Inferior Frontal Cortex Cortical Thickness**			
Turquoise	15.0	**Behavior and Cognition**	ADCY2 ATXN1 CACNA1A CACNB2 CADPS2 CTNND2 DACH1 GRID1 GRIN2A ITPR3 JAKMIP1 KCNJ4 LRRK2 NEGR1 NTRK3 PAK7 PARK2 PCLO PRKAR1B PRKCB SORCS3 STX1B TUSC3
		behavior, cognition, learning or memory	
		**Synaptic Transmission**	
		synaptic transmission	
**Right Inferior Frontal Cortex Cortical Thickness**			
Turquoise	13.0	–	–
Yellow	12.0	–	–
**Right Superior Temporal Gyrus Cortical Thickness**			
Turquoise	25.0	**Behavior and Cognition**	ATXN1 CACNA1A CACNA1C CNTNAP2 CTNND2 GABBR2 GRIN2A ITPR1 KCNB1 KCNJ4 KIRREL3 NALCN NAV2 NLGN1 NTRK3 PCLO PRKAR1B PRKCB SORCS3 SYT13 THRB
		behavior, cognition, learning or memory	
		**Synaptic Transmission**	
		synaptic transmission	
		**Metabolic Process**	
		insulin secretion	
**Functional Connectivity between Right Inferior Frontal Cortex and Left Inferior Frontal Cortex**			
Turquoise	15.0	**Behavior and CognitionBehavior, cognition, learning or memory, locomotion** Synaptic Transmission and SynaptogenesisSynaptic transmission, GABAergic synaptic transmission, glutamatergic synaptic transmission, glutamate receptor signaling pathway, cell surface receptor signaling pathway, transmembrane receptor protein tyrosine kinase signaling pathway, enzyme linked receptor protein signaling pathway, regulation of synaptic plasticity, neurotransmitter transport, regulation of transmembrane transporter activity, ion transmembrane transport, calcium ion transmembrane transport, regulation of small GTPase mediated signal transduction, regulation of alpha-amino-3-hydroxy-5-methyl-4-isoxazolepropionate (AMPA) selective glutamate receptor activity, N-methyl-D-aspartate (NMDA) receptor clustering, protein localization to synapse, cellular response to catecholamine, regulation of excitatory postsynaptic membrane potential	AAK1 ACSL6 ADCY8 AFF4 AFG3L2 AKTIP ALK ANK2 ANO2 ANTXR1 ANXA2 AQP9 ARHGEF3 ARNT ASH1L ASPH ATP2B2 ATXN1 BACH2 BAIAP2 BCL11B BTBD9 C11orf63 CA8 CACNA1D CACNA1I CACNG3 CADM2 CADPS CANT1 CASKIN1 CDC5L CDH2 CELSR1CHD9 CHERP CHL1 CHRNA3 CILP CMKLR1 CNGB3 CNIH3 CNKSR1 CNTN4 COBL COL19A1 CPT1A CRIM1 CTBP1 CTNNA2 CTSO CUX1 DACT2 DCC DDN DENND2A DGKI DGKZ DLC1 DLG2 DOCK4DOCK9 DPP6 DSCAM DSCAML1 DSP DYM EBF1 ELAVL2 ELMO1ERCC2 ERG FIG4 FOXN3 FOXO3 FRMD6 GABRB2 GABRG3 GFRA1 GNAL GPD2 GRIN2A GRM5 HECW1 HYOU1 IFIT1 IGF1R IL1RAP INPP5B ITGA9 ITSN2 JARID2 JPH4 KCND3 KCNIP4 KCNMA1 KCNQ3 KCNQ5 KCTD8 KDM3B KLF12 KLHL1 KRTAP5-9 KSR2 LDB2 LGI1 LMO2 LMTK2 LPCAT1 LPHN3 LRP8 LRRC4C LRRC8A LRRFIP1 LRRK2 LRRN1 LSAMP MACROD2 MAP2K4 MAP4K3 MARK3 MASTL MCTP1 MED12L MIB1 MMP17 MNAT1 MOCS2 MYH9 MYRIP NCAN NLGN1 NOS1 NPAS2 NPR3 NRG3 NRXN1 NUAK1NUMB OXR1 PACRG PARK2 PBX3 PCLO PDE10A PDE4D PDE7B PDZD8 PEX5L PHACTR1 PHACTR2 PIK3R1 PKD1L1 PLCB1 PLXNA4 PPP2R2B PRKAA1 PRKCE PRKG2 PTPRD PTPRE PTPRG PTPRJ PTPRN2 PTPRR PTPRT PXDN RAB31 RAPGEF4 RASA2 RBL2 RBMS3 RCAN1 RELN RFTN1 RNF168 ROBO2 RORA RPRD1A RPS6KA2 RTN4RL1 RYR2 SCOC SEMA5A SH3GL2 SIM2 SIPA1L2 SKAP1 SLC1A1 SLC22A3 SLC37A3 SLC6A1 SLC8A1 SLIT2 SMG6 SMOC2 SNTG1 SNX25 SOD2 SORCS1 SORCS3 SOX5 STAM STARD13 STAT4 STAT6 STK17A STYXL1 SV2B SYN3 SYNE1 TAF3 TBC1D13 TBC1D4TBC1D8 TFB2M THRB TIAM1 TNIK TRIM8 TTC8 TTN TTYH3 TUSC3 VAV3 VAX2 VEGFA VGLL4 VPS13A VWC2L XPR1 ZBTB46 ZC3H14 ZFPM2 ZMYM4 ZNF101 ZNF165 ZNF18 ZNF317 ZNF423 ZNF461 ZNF516 ZNF580 ZNF77 ZNF862 ZNF883
		**Brain Development and Organization**	
		Nervous system development, central nervous system development, anatomical structure development, brain development, neurogenesis, synapse organization, neuron differentiation, cell morphogenesis involved in neuron differentiation, stimulus neuron projection development, axonogenesis, axon guidance, dendrite development, axon extension involved in axon guidance	
		**Metabolic Process**	
		Regulation of metabolic process, phosphate-containing compound metabolic process, insulin-like growth factor receptor signaling pathway	
**Functional Connectivity between Right Inferior Frontal Cortex and Inferior Parietal Lobe**			
Turquoise	31.0	**Behavior and CognitionBehavior, cognition, learning or memory, locomotion, sensory perception of sound**	AAK1 ACSL6 ADAMTS16 ADCY9 ADIPOQ AFF4 ANK3 APP ARHGAP26 ARHGEF3 ASPH ATP2B2 ATP8A2 BCL11B BCL2L14 BTBD9 CA10 CACNA1D CACNA2D1 CACNB2 CADM2 CADPS CDC42BPA CDH13 CDH22 CHERP CHST11 CNR1 CNTN4 CNTN5 CNTNAP2 CRIM1 CRISPLD2 CTNNA2 DGKI DISP1 DLC1 DOCK3 DOCK4 DPPA2 DPP6 DSCAML1 EGFR EGLN1 EPB41L3 ERBB4 EVC EYA1 EYS FBXO11 FGF14 FMN2 FOXJ2 FOXP2 GABRG2 GABRG3 GCG GJB6 GNAL GPR55 GREM2 GRHL2 GRIN2A GRIP2 GRM1 HCN1 HDAC9 HMGA2 IGF1R IL1RN ITGA9 ITPR1 ITSN1 ITSN2 KALRN KCNA6 KCNAB1 KCNQ5 KCTD16 KIF20B KIF26B KIF5C KIRREL3 KRAS LMTK2 LPCAT1 LRP8 LRRC16A LRRK2 LYST MACF1 MAGI2 MAP2K4 MAPK14 MFGE8 MIDN MNAT1 MTOR MURC MYO10 NEGR1 NELL1 NLGN1 NOS1AP NRXN1 OLFM3 OPCML OSBPL8 PBX1 PCDH15 PDE10A PDE4D PDE8B PDGFRB PDZRN3 PEX5L PHF21A PLXNC1 PODXL PPFIA2 PPP1R16B PPP3CA PRKCB PTGIS PTPRD PTPRN2 PXDN RAB3C RASGEF1C RGS7 RIPK1 ROBO2 RPS6KA2 RPTOR RYR2 SCEL SCG5 SDCCAG8 SEC24D SEMA5A SEMA6D SERPINI1 SGIP1 SLC32A1 SLC6A1 SMAD3 SMYD1 SOX5 SPOCK1 SPTBN1 STAT6 STX1B SUFU SYN3 SYNJ1 SYT16 TACC2 TBR1 TDRD5 TECTA TGM1 THOC1 TNFSF11 TRAF5 TRIM8 TRPC6 TTBK1 TYR UNC13C USH2A VAV3 VPS13A VWC2L WNT3A XIRP2 YWHAE ZFP57 TRPV3
		**Synaptic Transmission and Synaptogenesis**	
		Synaptic transmission, GABAergic synaptic transmission, regulation of synaptic glutamatergic transmission, regulation of signaling, protein kinase A signaling, neurotrophin signaling pathway, neurotransmitter secretion, regulation of neurotransmitter levels, regulation of synaptic vesicle transport, regulation of calcium ion transport, regulation of potassium ion transmembrane transport, regulation of excitatory postsynaptic membrane potential, cellular response to catecholamine stimulus	
		**Brain Development and Organization**	
		Nervous system development, central nervous system development, brain development, anatomical structure development, neurogenesis, synapse organization, cell morphogenesis involved in neuron differentiation, neuron differentiation, neuron development, neuron projection development, neuron recognition, axon development, axon guidance, axonogenesis	

Among the 265 genes, 226 were expressed in the right IFC, which were significantly over-representative over this full set of genes (*p* < 0.001). Six modules were identified from these genes, including brown, turquoise, yellow, blue, green and grey (Fig. 4c **;** Preservation *Z*-scores ≥ 1.5) (Fig. 4c). Of the six modules, both brown and turquoise modules had the strongest Preservation *Z*-scores above 10. Brain-related biological processes were identified for both modules, i.e., positive regulation of axon extension involved in regeneration, neurogenesis, neuron projection development, dendrite development, and etc. But, none of these biological processes survived for the FDR correction of multiple comparisons. Likewise, GO biological processes for the other four modules also did not survive for the FDR correction of multiple comparisons (Table S2 in the Supplementary Material).

Similarly, among the 268 genes, 218 were expressed in the posterior region of STG, which was significantly over-representative over this full set of genes (*p* < 0.001). Four modules were constructed from these genes (turquoise, brown, blue, grey in Fig. 4b; Preservation *Z*-scores ≥ 2.3) of which the turquoise module had the highest Preservation Z-score of 12.0. This module largely reflected brain-related biological processes, such as behavior, cognition, learning or memory, synaptic transmission, and insulin secretion (Fig. 4b). Its major hub genes included GRIN2A, CACNA1A, CACNA1C CTNND2, PLCO, PRKCB, ITPR1, and etc (Table 3). For the other three modules, GO biological processes identified were less related to brain functions and did not survive for the FDR correction of multiple comparisons (Table S3 in the Supplementary Material).

### Biological processes for age by gene interaction on functional connectivity

Top 10% SNPs that most contributed to age by PRS_cross_ on the functional connectivity of the right IFC-left IFC (7638) and the functional connectivity of the right IFC-right IPL (5435) were mapped to 1871 genes and 1417, respectively.

**Fig. 5:**
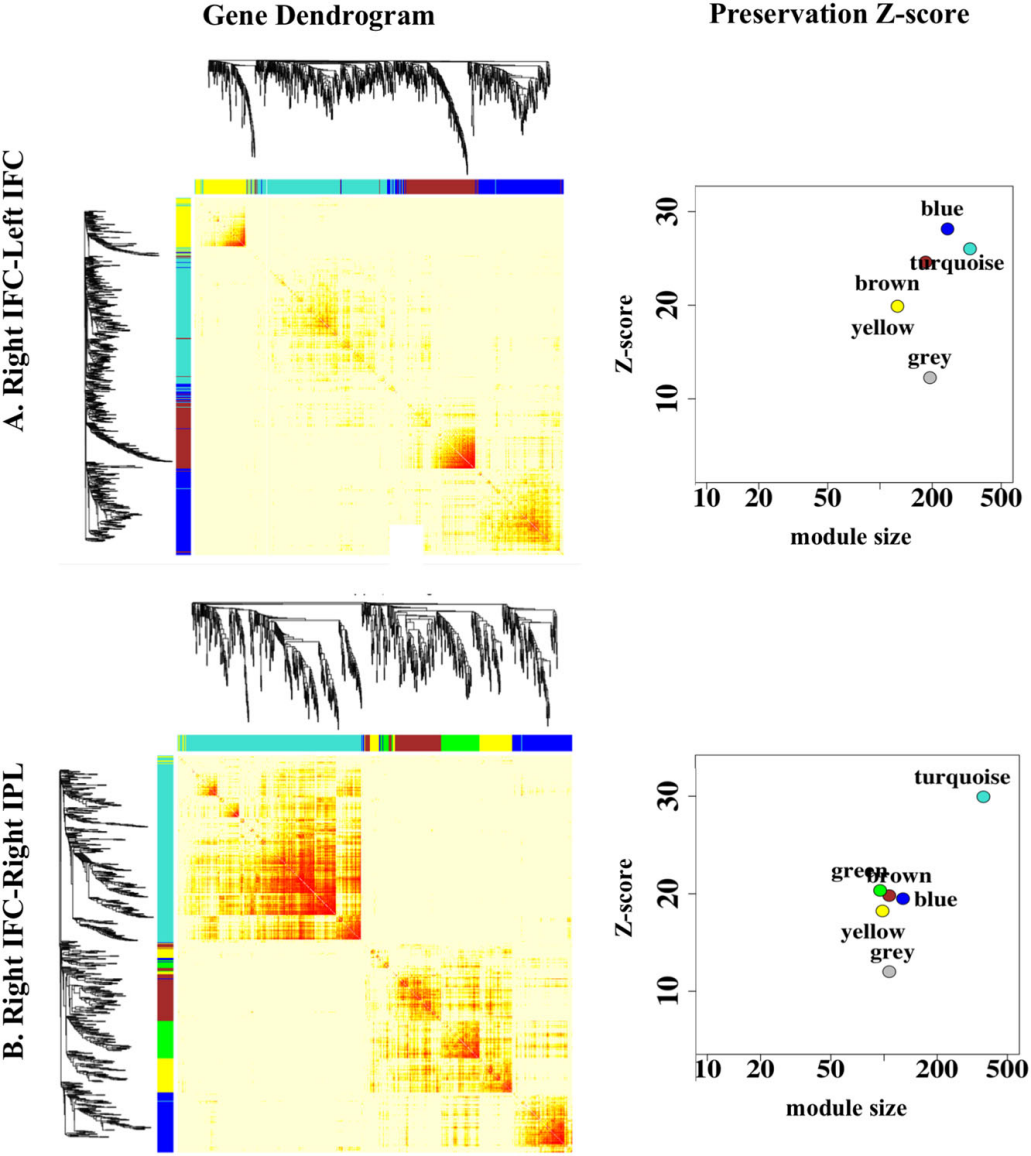
Genetic modules. The first column shows the WGCNA gene dendrogram for shared genes between bilateral inferior frontal cortex (IFC; top) and between IFC and right inferior parietal lobe (IPL; bottom). The second column illustrates the robustness of each module as indicated by the Preservation Z-score

Assuming that genes with common expression profiles between two cortical regions underlie the molecular basis for the functional organization. Genes with similar expression patterns between right IFC and left IFC as well as between right IFC and left IFC were identified via maxT, Kolmogorov–Smirnov test (KS test) and Radial Kolmogorov-Smirnov test (RKStest) analyses. Gene expression profiles of these regions were obtained from the Allen Brain Atlas gene transcriptome data. For the functional connectivity of right IFC-left IFC, 1505 out of 1871 genes were commonly expressed in both left and right IFC. Nevertheless, maxT, KStest and RKStest analyses only revealed 1078 genes expressed in bilateral IFC at the same level of gene expression and its variance (*p* > 0.05). These 1078 genes were significantly over-representative over this full set of genes (*p* < 0.001). Five genetic modules were further identified using WGCNA (Fig. 5a, Preservation *Z*-scores ≥ 12.0), namely, blue, turquoise, brown, yellow, and grey modules. Of note, genetic network structures among these genes were much more robust than those seen in Fig. 4 with turquoise module showing a strong Preservation *Z*-score of 25.0. This module largely reflected brain-related biological processes such as behavior, cognition, learning or memory, brain development, neuron projection development, synaptic transmission, GABAergic synaptic transmission, glutamate receptor signaling pathway, insulin-like growth factor receptor signaling pathway, regulation of synaptic plasticity and synapse organization (significant FDR corrected p-values reported in Table S4 of the Supplementary Material). The major hub genes of this module comprised GRIN2A, GRM5, CTNND2, CACNA1D, PLCO, PRKCE, ROBO2, SLIT2, RELN, GABRG2, SLC6A1, IGF1R, and etc (Table 3). For the other 4 modules, GO biological processes were less related to brain and none of them survived for the FDR correction of multiple comparisons (Table S4 in the Supplementary Material).

Likewise, for the functional connectivity of the right IFC-right IPL, 1150 out of 1417 genes were commonly expressed in both right IFC and right IPL. However, only 901 genes were not differentially expressed in the right IFC and right IPL (*p* > 0.05), which was significantly over-representative over this full set of genes (*p* = 0.0035). These 901 genes formed six modules using WGCNA, including turquoise, green, brown, blue, yellow and grey modules (Preservation *Z*-scores ≥ 10.0; Fig. 5b). Among the six modules, the turquoise module had strongest Preservation *Z*-scores of 31.0 and was most reflective of brain-related biological processes, such as cognition, learning or memory, nervous system development, neuron projection development, brain development, synaptic transmission, GABAergic synaptic transmission, regulation of glutamatergic synaptic transmission, synapse organization and neurotrophin signaling pathway (significant FDR corrected *p*-values reported in Table S5 of the Supplementary Material). The major hub genes of this module comprised of GRIN2A, GRM5, CTNND2, CACNA1D, PRKCB, ROBO2, SLIT2, GABRG2, SLC6A1, APP, and etc (Table 3). The blue and green modules with Preservation *Z*-scores of 17.0 and 19.0 respectively also showed significant biological processes after the FDR correction for multiple comparisons. However, most of the biological processes associated with these two modules were non-brain related (significant FDR corrected p-values reported in Table S5 of the Supplementary Material). GO biological processes of the other three modules were also less related to the brain and did not survive for the FDR correction of multiple comparisons (Table S5 in the Supplementary Material).

## Discussion

This study examined whether the polygenic risk for psychiatric disorders modulated age-related alterations in cortical thickness and its corresponding functional connectivity using a Chinese sample comprised of adults aged from 21 years and above. Our results showed age-related cortical thinning in bilateral IFC and STG and alterations in the functional connectivity between bilateral IFC and between right IFC and right IPL as a function of the polygenic risk for psychiatric disorders. The genes, that were involved in this polygenic risk and contributed most to age-related alterations in cortical thickness and functional connectivity, were expressed in the corresponding cortical regions and had biological processes related to neural plasticity, synaptogenesis and metabolism. Especially, genes identified through the genotype-functional connectivity association analysis were commonly expressed in both cortical regions and formed strong gene networks contributing to neural plasticity, synaptogenesis, and metabolism, suggesting convergent evidence on potential candidate molecular mechanisms for aging brain.

Our findings underlined a modulative role of psychiatric risk genes in aging brain, particularly in cortical regions that have been considered as hallmarks of normal aging and psychiatric disorders. The IFC and STG show pronounced age-related cortical thinning^11,12,51^. Likewise, individuals with psychiatric disorders, such as schizophrenia and bipolar disorders, show cortical thinning in these brain regions as compared to healthy adults^52–56^. In parallel with brain morphology, functional connectivity between bilateral IFC and between right IFC and IPL are also found to be susceptible to both normal aging and psychiatric disorders^14,15,57^. Taken together, these findings suggest that brain morphology and functional organization commonly vulnerable to aging and psychiatric disorders are as a function of the polygenic risk for psychiatric disorders.

Our findings suggested antagonistic pleiotropy, that is, impact of certain genes may differ at different stages of life. By far, this phenomenon has been substantially supported by the findings of APOE and COMT on behavioral and neural endophenotypes across lifespan^25,58–64^. Apparently, this phenomenon was also supported by our findings related to the polygenic variants for psychiatric disorders. Neural benefits that are genetically programmed by polygenic risk genes for psychiatric disorders include thicker cortex and stronger functional connectivity in early life but with a potential bearing towards neural susceptibility in late life. Nevertheless, our findings showed that older adults with a lower polygenic risk was associated with greater age-related cortical thinning and reduction in the functional connectivity as compared to those with a higher polygenic risk. This is opposite to what is shown in Caucasian populations^65,66^, which underscores the importance of population differences in genetic influences on imaging phenotypes.

This study showed shared biological processes contributing to age-related cortical thinning in bilateral IFC and right STG and reduction in their functional connectivity. These processes involve biological functions related to behaviors, cognition, learning and memory, synaptic transmission, and metabolism. These biological functions may highlight potential molecular mechanisms for understanding the role of IFC morphology and functional organization in an age-related decline in memory and learning^67,68^. First, this study highlighted a set of genes (GRIN2A, CACNA1A, CACNA1C, CTNND2, PLCO, PRKC, GRM1, GRM5, etc.). These genes implicated long-term potentiation (LTP) as one of their major functions. LTP is a form of activity-dependent plasticity that results in a persistent enhancement of synaptic transmission^69^. LTP requires coincident detection of pre- and post-synaptic depolarization, which is accomplished through the NMDA receptor, a voltage-dependent subtype of glutamate receptor that allows permeation of calcium and other cations. In addition, there exist a wide range of other forms of LTP that is induced through activation of the metabotropic glutamate receptor^70^. Indeed, experimentally removing or altering certain genes in mice, such as CACNA1C, GRIN2A and GRM5, modifies LTP, which results in a long-lasting increase in the strength of synaptic transmission^71–74^. Even though these molecular mechanisms of LTP were initially discovered in the hippocampus, they exist at these synapses and throughout the brain. It has been demonstrated that LTP-related changes in synaptic strength occur as memory is formed at various sets of synapses in the brain^75^. LTP-synaptic plasticity in the prefrontal cortex is modified due to aging^76–78^. Acetylcholine, one of major available pharmaceutical treatments for Alzhermier’s disease, targets to improve memory function via enhancing the effects of LTP induction^79^. Complementary to animal and drug research mentioned above, this study employed brain imaging and bioinformatics techniques and provided new evidence on a potential fundamental molecular mechanism of LTP in age-related alterations in IFC thickness and its functional organization.

Second, this study also identified a set of genes (IGF1R, PIK3R1, PLCB1, ITPR1, CACNA1C, etc), with insulin secretion and insulin-like growth factor signaling functions, in link with age-related cortical thinning of the temporal cortex and reduction in the functional connectivity of the prefrontal cortex. There have been great efforts to link the brain insulin/IGF1 with neuropathologies mainly because canonical signaling of their receptors, including the phosphoinositide 3‑kinase (PI3K)–AKT–forkhead box protein O (FOXO) and RAS–mitogen-activated protein kinase (MAPK) pathways, influences apoptosis, neuronal antioxidant defence, tau phosphorylation, neuronal survival, and synaptic transmission^80,81^. Insulin seems to protect against the toxic effects of α-amino-3-hydroxy-5-methyl-4-isoxazolepropionic acid receptor (AMPA), oxygen/glucose deprivation and to prevent apoptosis^82^. Losing the protective effects of insulin, the brain may be at an increased risk for neurodegeneration. IGF1 is also suggested to play a beneficial role in neurodegenerative processes (1) by its potent pro-survial effects on damaged neurons through the PI3K-ATK pathway^83^; (2) by promoting amyloid clearance through blood-brain-barrier for the protection against Alzheimer’s pathology^84^; and (3) by blocking the action of inflammatory cytokines for protecting neurons against maladaptive inflammation that is a common process underlying in neurodegeneration^85^. Moreover, both insulin and IGF1 influence learning and memory by modulating neuronal plasticity. They modulate LTP, long-term depression, and changes in synaptic strength by changing transmission and synthesis of glutamate and GABA receptors though the PI3K-ATK pathway and modulate neuronal excitability by affecting ion-channels through MAPK pathways^83^. Disruptions of IGR1R and CACNA1C in animal models are associated with enhanced apoptosis, reduced neuronal survival rate, and lower glucose metabolism^80,86,87^. Intriguingly, glutamate or amyloid peptides interfere with IGF1R signaling, leading to a state of IGF1 resistance in Alzheimer’s disease, a trait that may profoundly affect its course^84^. Clinical trials with insulin show improved cognition in patients with Alzheimer’s disease^88^. Although it is unclear why the metabolic functions only targeted to aged-related brain atrophy in the temporal cortex and functional disconnection in the frontal cortex shown in this study, the spatial distribution of the insulin and IGF1 in the brain are highly dependent on the availability of glucose and lipids to brain centers involved in energy allocation. Substantial evidence from FDG-PET/fMRI-studies suggests an association between glucose consumption and functional connectivity in the frontal region^89,90^. The rest-state glucose consumption is remarkably correlated with the distribution of amyloid plaques^91^. Moreover, dysregulation of glucose metabolism, such as hypoglycemia, has been found to be associated with thinning of the superior temporal and prefrontal cortex^92,93^. Notwithstanding, hypoglycemia is also associated with decreased prefrontal functional connectivity within the default-mode network and frontal-parietal network^94,95^. These findings highlight possible “metabolism hypothesis” for aging and Alzheimer’s disease.

The most intriguing finding in this study demonstrated that the genes contributing to age-related alterations in the frontal functional connectivity formed much more robust gene networks (all Preservation *Z*-score > 10) than those observed in cortical morphology, supporting the idea on common molecular bases for age-related alterations in the functional communication between two brain regions. These molecular functions included synaptic transmission, neurotrophin signaling pathway, synaptogenesis, and metabolism. Again, the genes (GRIN2A, GRM1, PLCO, IGF1R, PIK3R1, PLCB1, etc), identified based on the frontal functional connectivity, largely overlapped with those derived from the frontal cortical morphology. As mentioned earlier, they are involved in the glutamatergic and GABAergic transmission and metabolic processes that regulate neural plasticity important for learning and memory^83^. Indeed, reduced GABAergic signaling is associated with less IFC engagement during learning task^96^. Age-related dysregulation of GABAergic signaling in IFC contributes to working memory impairment in aging^97^. Decline in glutamate neurotransmitter is associated with dysregulated synaptic plasticity and impaired working memory^98^. Likewise, the neurotrophin signaling pathway, comprised of MAPK14, PIK3R1, FOXO3, ITPR1, VAV3, EGFR, ERBB4, PRKCE, ADCY9, KRAS, and etc, involves a family of trophic factors that contribute to differentiation and survival of neural cells. The neurotrophin signaling is regulated by connecting a variety of intracellular signaling cascades, which include MAPK pathway, PI3K–pathway, and PLC pathway, and transmits positive signals to enhance neural survival and growth. Hence, this signal plays an important role for neural development, neuroprotection against age-related cellular insults, neural plasticity, and higher-order activities such as learning and memory^99,100^. Decreases in the expression of MAPK, neurotrophin and their receptors are associated with advanced aging^101–103^. Most importantly, neural metabolism, synaptic transmission, neurotrophin are all critical for synaptogenesis. This study showed that genes (VAV3, ROBO2, SLIT2, RELN, IGF1R, CACNA1D, CACNA1I, etc) involve in numerous biological processes, including dendrite development, axonogenesis, and neuron projection, closely associated with synaptogenesis. The establishment of precise connections among neurons usually requires a balance between excitatory and inhibitory synapses. Both glutamatergic and GABAergic transmission mediate dendritic growth, arbor and length during synaptogenesis^104,105^, which shapes the cortical circuitry during brain development^105,106^. Likewise, neurotrophin factors regulate several functions for synapse formation, including enhancement of transmitter release and increased concentration of vesicles. Furthermore, both insulin and IGF1 regulate dendritogenesis, synapse maintenance, and axon guidance through PI3K–AKT signaling^107^. IGF1 involves in the production and migration of GABAergic and glutamatergic neurons and thus contributes to the formation of appropriate circuitries in the cortex^108^. All together support the idea on age-related reduction in capacity of synaptogenesis^109^.

This study is best considered as preliminary and exploratory and intended to provide a strategy for the analysis of gene–age interdependence and identify candidate biological processes that might mediate the influence of aging associated with the genetic risk for psychiatric disorders on neurodegeneration. While combining genotype and transcriptome data with multi-modal brain images is highly unique, it is nevertheless a modest sample size for genomic analyses. However, our findings do suggest biologically plausible candidate processes for age-related degeneration in both brain morphology and functional organization. Especially, genes modulating the age-related functional dysconnectivity are well expressed in the functionally connected brain regions and form strong genetic networks contributing to synaptic transmission and synaptogenesis.

In conclusion, this preliminary study suggests the feasibility to identify biological mechanisms for aging brain via integrating genotype and transcriptome with neuroimage data. Our findings suggest that neural plasticity and synaptogenesis, particularly regulated by glutamatergic and GABAergic transmission, neurotrophin signaling, and neural metabolism, are core biological mechanisms contributing to age-related cortical thinning and functional disconnection.

## Electronic supplementary material

Supplementary tables
